# Defining and reporting activity patterns: a modified Delphi study

**DOI:** 10.1186/s12966-023-01482-6

**Published:** 2023-07-25

**Authors:** Nicola D. Ridgers, Emily Denniss, Alissa J. Burnett, Jo Salmon, Simone J.J.M. Verswijveren

**Affiliations:** 1 Allied Health and Human Performance, Alliance for Research in Exercise, Nutrition and Activity (ARENA), University of South Australia, Adelaide, South Australia Australia; 2 Institute for Physical Activity and Nutrition (IPAN), School of Exercise and Nutrition Sciences, Deakin University, Geelong, VIC Australia

**Keywords:** Physical activity, Sedentary behaviour, Movement behaviours

## Abstract

**Background:**

Despite significant interest in assessing activity patterns in different populations, there has been no consensus concerning the definition and operationalisation of this term. This has limited the comparability, interpretability, and synthesis of study findings to date. The aim of this study was to establish a consensus regarding the way in which activity patterns and activity pattern components are defined and reported.

**Methods:**

The activity patterns literature was searched to identify experts to be invited to participate and to develop a proposed definition of activity patterns and activity pattern components. A three-round modified Delphi survey was conducted online (November 2021 to May 2022). In Round 1, participants were asked to rate their agreement with a proposed activity patterns definition, which also included six activity pattern components (e.g., activity intensity, activity bout, transitions), six examples of activity patterns (e.g., frequency of postural transitions in discrete time periods) and eight items for reporting activity patterns in future research (n = 21 items). Open-ended questions enabled participants to provide further comments and suggestions for additional items. Consensus was defined a priori as ≥ 80% participants rating their agreement with an item. In Round 2, participants were asked to rate their agreement with 25 items (13 original items, eight amended, and four new). In Round 3, participants rated their agreement with 10 items (five original items, four amended, and one new).

**Results:**

Twenty experts in activity patterns research participated in Round 1, with response rates of 80% and 60% in Rounds 2 and 3, respectively. The proposed activity pattern definition, all activity pattern components definitions, four of the six activity pattern examples, and 10 items in the activity patterns reporting framework achieved consensus. The removal of one activity component item between Rounds 1 and 2 achieved consensus.

**Conclusion:**

This modified Delphi study achieved consensus for defining and reporting activity patterns for the first time. This consensus definition enables standardisation of activity patterns terminology, which is important given the significant interest in quantifying how individuals accumulate their physical activity and sedentary behaviour across the lifespan to inform the development of future public health guidelines and interventions efforts.

**Supplementary Information:**

The online version contains supplementary material available at 10.1186/s12966-023-01482-6.

## Introduction

Physical activity is important for physical, mental, and cognitive health across all ages [1–5]. In contrast, emerging evidence shows that excessive sedentary behaviours (such as recreational screen time) have detrimental impacts on population health [6–9]. Recently released global [10] and national [11] 24-hour movement guidelines encourage optimal combinations of physical activity, sedentary behaviour, and sleep to benefit health. Nevertheless, these recommendations do not include advice on how to specifically accumulate physical activity and sedentary behaviour throughout the waking day. For example, it is unclear whether accumulating physical activity sporadically is healthier than less frequent but sustained periods, or vice versa. As some emerging evidence suggests that these “activity patterns” may be important for child [12, 13] and adult [14] health, it is important to further investigate whether such recommendations should be provided in the future.

Despite the increased interest in the relationship between activity patterns and health outcomes, there is a lack of consistency in results obtained from such studies that limits the development of guidelines. As highlighted in a systematic review by Gomes and colleagues [15], this may be due to the complexity that comes with attempting to quantify and understand activity patterns [16]. The total volume of physical activity and sedentary behaviour can be accrued in an array of diverse ways, including varying frequencies, intensities and duration of activity bouts (e.g., at least 10-min in moderate- to vigorous-intensity physical activity) and postural transitions (e.g., sit-to-stand transitions). Such complexity is further compounded by the setting (e.g., school, home, community), segment (e.g., school hours versus out-of-school hours), type of day (weekday versus weekend day) and seasons, amongst other factors. Consequently, few studies have used consistent terminology and definitions when investigating activity patterns. This is evident from a previous systematic review that focused on activity patterns and cardiometabolic risk factors in youth, which concluded that it was difficult to draw conclusions due to the substantial heterogeneity in pattern definitions [17]. When focusing on the accumulation of activity, the review showed that bout lengths ranged from ≥ 4-seconds to ≥ 20-minute bouts for physical activity and ≥ 1-minute to ≥ 2-hour bouts for sedentary behaviour [17]. Inconsistent definitions can make it difficult to agree on what is being researched and may lead to studies examining disparate or heterogeneous concepts that can limit comparability between studies and hinder the advancement of the evidence base [18]. It is therefore critical to establish a consensus for how activity patterns and activity pattern components should be defined and consistently reported in the literature, to enable the interpretability, comparability, and synthesis of activity patterns research.

The Sedentary Behavior Research Network provided some clarity with regards to sedentary patterns through a Terminology Consensus Project [19]. Upon reviewing the literature and addressing feedback from members, a consensus definition for sedentary patterns was obtained: “the manner in which sedentary behaviour is accumulated throughout the day or week while awake (e.g., the timing, duration and frequency of sedentary bouts and breaks)” [19]. However, this definition does not capture the entire activity spectrum, including physical activity, and while some examples of pattern components were described (i.e., bouts, breaks), no guidance on how activity patterns should be reported was provided. To compare future studies assessing activity patterns and replicate studies in different populations, standardised pattern definitions and reporting of activity patterns are critical.

One widely used research tool to reach consensus is the Delphi method [20]. The Delphi method is suitable for developing new concepts, definitions, and tools, and has been frequently used in health research [18, 21–24]. This method provides an opportunity to come to an agreement on a definition of activity patterns as well as develop a framework for reporting activity patterns in the literature. Therefore, the aim of this study was to develop a consensus regarding the way in which activity patterns and pattern components are defined using the Delphi method. A secondary aim was to develop a consensus for a framework for reporting activity patterns research.

## Methods

### Study design

This study utilised a modified Delphi method, which is a flexible approach for gaining views of experts and research consensus via iterative surveys and controlled feedback [21, 25]. It is recommended that the number of Delphi surveys is determined *a priori*, and three rounds of surveys is considered optimal [21]. Therefore, three rounds were set *a priori*. Data were collected between November 2021 and May 2022. Qualtrics (Qualtrics, Provo, UT) was used to collect survey data. REDCap (Vanderbilt University, TN) was used to store and manage data.

### Participants

The activity patterns literature was searched using the PubMed database to identify national and international academics with expertise in physical activity and/or sedentary behaviour patterns. Relevant search terms related to physical activity and sedentary behaviour patterns were identified from previous systematic reviews [15, 17]. A list of authors (first, senior) who had published at least one paper on the topic of physical activity or sedentary behaviour patterns, and whose email addresses were publicly available, was created. The Delphi literature recommends that 10 to 15 people is an appropriate sample size where a sample is homogenous [21, 24]. Aiming for global representation and accounting for potential participant drop-out, 26 researchers were identified and received an individualised email invitation to participate and a link to the first survey. Snowball sampling was also used and a request to forward the study information to others with relevant expertise was included in the email invitation. Participants provided informed voluntary consent at the start of the first online survey. Up to two email reminders were sent to participants prior to the survey completion deadline if no response was received. Ethical approval was obtained from Deakin University Human Ethics Advisory Group – Health (HEAG-H 181–2021).

#### Survey development and pilot testing

A search of existing literature was conducted in PubMed to identify studies, including systematic reviews, that had examined patterns of physical activity and/or sedentary behaviour. Studies published since 2010 until June 2021 were investigated. The literature search focused on identifying (a) activity pattern definitions that had previously been used, and (b) the ways in which specific activity pattern components were operationalised and defined. Examples of activity patterns were also extracted. Lastly, a framework was developed for reporting activity patterns in the published literature. The information collated through the literature search was discussed by the authorship team in group meetings (August 2021) and a proposed definition of activity patterns and specific components of patterns were drafted until agreement was achieved. Components of the proposed reporting framework were also identified and discussed by the authors. Activity pattern examples were selected for inclusion in the Round 1 survey to reflect the different ways in which patterns have been examined in the literature. The Round 1 survey was piloted with academics (n = 3; based in Australia) with expertise in physical activity and sedentary behaviour to determine the readability of the developed statements and to improve the survey structure and clarity of instructions provided. Minor changes were suggested concerning the wording of instructions, but no changes were made to the statements used in the survey.

### Round 1 survey

The Round 1 survey consisted of three sections. In section one, participants were asked to provide details about their professional background, such as role, area of expertise, number of years working in their field, and country of employment. In section two, participants were presented with a proposed definition of activity patterns, definitions of six components of activity patterns (e.g., posture, activity bout, etc.) and six examples of activity patterns (e.g., frequency, intensity, and duration of activity bouts that occur throughout the day). Participants were asked to rate their agreement with each definition or example on a five-point Likert scale, ranging from 1) strongly agree to 5) strongly disagree. A five-point scale was chosen because Likert-scales are considered optimal for rating statements in Delphi research and it is recommended that scales include between four and seven options [21, 26]. Additionally, three open-ended questions were included so that experts could provide comments or suggestions about the proposed conceptual definition of activity patterns, definitions of specific activity pattern components, and examples of activity patterns. In section three, a proposed framework for reporting activity patterns in the literature was provided. Eight statements for reporting activity patterns research (e.g., the activity intensity [or intensities] and/or posture(s) being investigated should be clearly defined and reported) were based on existing literature and participants were asked to rate their importance on a five-point Likert scale ranging from 1) very important to 5) not at all important. An open-ended question was also included so that experts could suggest additional components to the framework or provide comments on the proposed items.

Where free text responses were provided in the Round 1 survey, these were read by the research team and discussed in relation to the overall responses to the survey statements. Whilst it was not a requirement for multiple participants to make similar recommendations for an item to be modified or added, recommendations made by multiple participants were weighted more heavily in the discussions when addressing the recommendations made and finalising any changes to items in Round 2.

### Round 2 survey

**Fig. 1:**
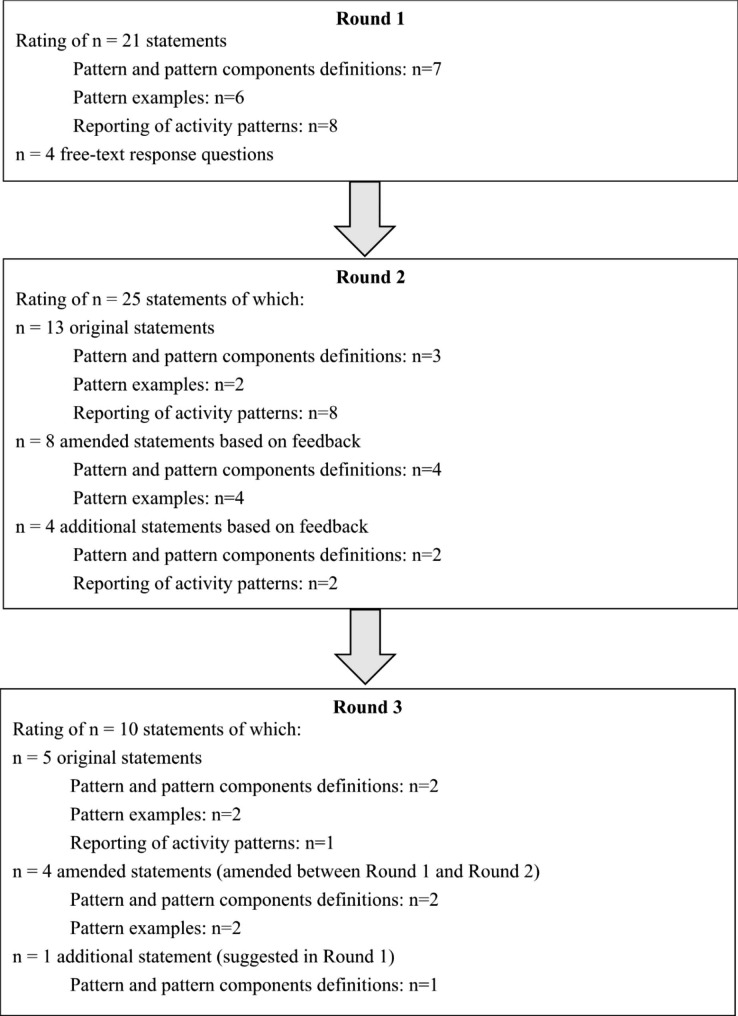
A flow diagram presenting the Delphi study process

The Round 2 survey consisted of two sections. In section one, participants were presented with an updated definition of activity patterns, seven operational definitions of activity pattern components, and six examples of activity patterns. The activity patterns definition was updated based on feedback provided from the open-ended questions and subsequent discussion amongst the authors. Seven (as opposed to six in the Round 1 survey) components of activity patterns were defined, as two additional components were suggested by participants in response to the open-ended question in Round 1, and one component was removed based on participant feedback. Four of the six examples of activity patterns were revised to improve clarity based on participant feedback and two were the original statements that were updated for consistency with the revised examples (see Fig. 1). Section two in the Round 2 survey focused on the activity patterns reporting framework and included the same original eight components of the reporting framework from Round 1. Lastly, two additional components suggested by participants in response to Round 1 were also included. Experts were asked to rate the importance of each component of the reporting framework on the five-point scale.

Summaries of the Round 1 verbatim responses to the open-ended questions and Likert-scale responses were made available to participants when responding to the Round 2 survey. Changes made to items within the Round 2 survey were identified to participants for transparency. Feedback about responses to Likert-type scale questions from Round 1 were presented in a bar graph and included the median and interquartile range of participants’ responses, as recommended in the Delphi literature [21, 26]. When responding to questions, participants were asked to consider the responses from the rest of the group when formulating their opinion, to encourage consensus [21].

### Round 3 survey

The Round 3 survey followed the same format as the Round 2 survey alongside corresponding feedback about the group responses to the Round 2 survey. Participants were again asked to consider the responses from the rest of the group when responding to the Round 3 survey, which were presented in a bar graph and with the median and interquartile range provided. Statements that achieved consensus in Round 2 were not included in the Round 3 survey. No adaptations to the proposed definitions, examples or reporting framework statements occurred between Rounds 2 and 3. In section one, the definition of activity patterns and four definitions of activity patterns components were presented (others reached consensus in Round 2). In section two, one component of the reporting framework was presented as the rest of the components reached consensus in Round 2.

### Data analysis

Descriptive statistics (proportions) were used to describe participants’ demographic characteristics and responses to each statement within the surveys. In this study, responses to strongly agree and agree were combined to create an ‘agree’ category, while responses to strongly disagree and disagree were combined to create a ‘disagree’ category [27]. For the importance ratings, ‘very important’ or ‘important’ were combined to create an ‘important category’, while ‘unimportant’ or ‘not important at all’ were combined to create an ‘unimportant’ category. In this study, consensus was defined as ≥ 80% of participants selecting ‘agree’ or ‘important’, or ‘disagree’ or ‘unimportant’ on the Likert scale and was determined *a priori*. Whilst there is no agreed upon figure for consensus in the Delphi literature, 80% has been suggested as an appropriate cut point in health research [24]. In Delphi research, stability of consensus is most often evidenced through increasing weighted Kappa values, which was not possible in the present study due to the modification of survey statements between the Round 1 and Round 2 surveys. Therefore, stability of consensus was not calculated in this study. Similar approaches have been taken in previous Delphi studies that adapted survey statements between rounds [23, 27].

## Results

In total, 20 experts participated in Round 1 of this Delphi study, with 17 recruited via the email invitation and three recruited through snowballing. Of these participants, 16 (80%) and 12 (60%) completed Round 2 and Round 3, respectively. Participant characteristics are provided in Table 1. Overall, participants were from four continents/regions, and 55% had > 10 years of research experience in this field.

**Table 1:** Demographics characteristics of participants

	Round 1 (n = 20) n (%)	Round 2 (n = 16) n (%)	Round 3 (n = 12) n (%)
*Continent/Region of residence*			
North America	5 (25)	2 (13)	1 (8)
South America	1 (5)	1 (6)	1 (8)
Europe	12 (60)	11 (69)	9 (75)
Oceania	2 (10)	2 (13)	1 (8)
*Current role*			
Professor	5 (25)	5 (31)	4 (33)
Associate Professor	6 (30)	6 (38)	4 (33)
Lecturer	2 (10)	-	-
Research Fellow	5 (25)	3 (19)	2 (17)
PhD student	2 (10)	2 (13)	2 (17)
*Years working in the field*			
20 + years	5 (25)	5 (31)	4 (33)
15–20 years	2 (10)	2 (13)	-
10–15 years	4 (20)	4 (25)	3 (25)
5–10 years	6 (30)	3 (19)	3 (25)
0–5 years	3 (15)	2 (13)	2 (17)

### Round 1

In Round 1, the survey consisted of 21 statements (activity patterns and components = 7 statements; activity pattern examples = 6 statements; activity patterns reporting framework = 8 statements) and 4 open response statements. Table 2 provides a summary of the responses to the initially developed definition of activity patterns, the definitions of six activity pattern components, and the six examples of activity patterns. Detailed information about the original definitions presented in Round 1 are provided in Supplementary Table 1. Consensus was not achieved for the definition of activity patterns in this round. Free text responses about the definition of activity patterns were provided by 70% participants, with suggestions for including movement behaviours and how to incorporate different time scales within the definition. Consensus was achieved for two activity pattern component definitions, which were activity bout (80%) and transition (90%). Supporting free text responses for some of the activity pattern components was provided by 70% of participants, and primarily focused on suggestions for refining specific activity pattern components and how activity is accumulated. Consensus was not achieved for any of the provided activity pattern examples.

**Table 2:** Results from Round 1 for activity pattern definitions, components, and examples

	Responses (n = 20)	Agreement (%)*
***Activity patterns and components definitions***		
Activity patterns definition	20	45%
Activity intensity	20	50%
Posture	20	75%
How activity is accumulated	20	45%
Activity bout	20	**80%**
When activity is accumulated	19	63%
Transition	20	**90%**
***Activity pattern examples***		
Frequency, intensity and duration of activity bouts that occur throughout the day (e.g., minutes spent in 20-min sedentary bouts).	20	75%
Total volume of at least one intensity accumulated in discrete time period(s) during the day (e.g., recess, lunchtime, after school).	20	45%
Frequency of postural transitions in discrete time period(s) during the day (e.g., at work, during class time).	20	70%
Total volume of at least one intensity accumulated across different seasons (e.g., winter, summer).	20	45%
Frequency, intensity and duration of activity bouts accumulated on different days of the week (e.g., weekday vs. weekend day).	20	65%
Frequency, intensity and duration of activity bouts accumulated on different days of the week (e.g., weekday vs. weekend day).	20	70%

* Percentage of participants reporting ‘Strongly agree’ or ‘Agree’ (Definitions, Pattern examples) or ‘Very important’ or ‘Important’ (Reporting framework)

Note: Bold % = 80% consensus achieved.

There was consensus that all eight statements included in the reporting activity patterns research framework were important (see Table 3). Free text responses were provided by 40% of participants, with additional statements (e.g., “The processing of activity patterns data should be clearly reported”) and examples concerning the proposed statements being suggested.

**Table 3:** Results from Round 1, Round 2 and Round 3 for the Activity Patterns Reporting Framework

Statement	Round 1 (n = 20)		Round 2 (n = 16)		Round 3 (n = 12)	
	Important (%)	Not important (%)	Important (%)	Not important (%)	Important (%)	Not important (%)
The activity intensity (or intensities) and/or posture(s) being investigated should be clearly defined and reported	**95%**	0%	**100%**	0%	-	-
An explanation of how specific activity pattern components are defined/derived should be clearly reported	**100%**	0%	**93.4%**	0%	-	-
A rationale for examining activity bout(s) and/or transitions should be reported, where applicable	**85%**	0%	75%	6.3%	**100%**	0%
The way in which activity bouts and/or transition data are defined and analysed should be clearly reported, where applicable	**95%**	0%	**93.8%**	0%	-	-
The time period(s) and/or days of interest should be clearly defined, where applicable.	**100%**	0%	**93.8%**	0%	-	-
A rationale for the choice of any specific time period(s) and/or days of interest should be clearly provided.^2^	**85%**	0%	75%	6.3%		
The outcome variables for the time period(s) and/or days should be clearly reported	**95%**	0%	**93.8%**	0%	-	-
The method used to assess activity patterns should be clearly reported	**100%**	0%	**100%**	0%	-	-
The processing of activity patterns data should be clearly reported^1^	**-**	-	**100%**	0%	-	-
A rationale for choosing and defining specific activity pattern components should be reported, where applicable^1^	**-**	-	**87.5%**	0%	-	-

*Percentage of participants reporting ‘Very important’ or ‘Important’

** Percentage of participants reporting ‘Unimportant’ or ‘Not at all important’

^1^ New statement in Round 2

^2^ Statement missing from Round 3 survey due to technical error

### Round 2

The Round 2 survey consisted of 25 statements (activity patterns and components = 9 statements; activity pattern examples = 6 statements; activity patterns reporting framework = 10 statements). Four new statements were included, and four amended statements based on participant feedback were provided (Supplementary Table 1).

Consensus was not achieved in Round 2 for the revised activity patterns definition based on participant feedback. Consensus (≥ 80%) was obtained for 13 statements (see Table 4). Three of these statements (activity intensity: 88%; activity bout: 81%; and frequency: 88%) were for activity pattern components; two for activity pattern examples, and eight for the activity patterns reporting framework. Of note, the removal of the component “How activity is accumulated” from the activity pattern components also reached consensus (88%).

**Table 4:** Results from Round 2 and Round 3 for activity pattern and component definitions and examples

		Round 2 (n = 16)		Round 3 (n = 12)	
		Agree (%)*	Disagree (%)**	Agree (%)*	Disagree (%)**
***Final definition of activity patterns and activity pattern components***					
Activity patterns^1^	The temporal structure of physical activity and sedentary behaviour [movement behaviours] accumulated over a specified time period during the waking hours	62.5%	12.5%	**83.3%**	8.3%
Activity intensity^1^	Rate of energy expenditure required to perform waking activities	**87.5%**	6.3%	-	-
Posture^1^	The posture of the body (e.g., lying, reclining, sitting, or upright)	75%	12.5%	**91.7%**	8.3%
Activity bout	Unbroken period of time engaged in physical activity and/or sedentary behaviour ([33, 37])	**81.3%**	6.3%	**-**	**-**
Transition	Change from one activity intensity or posture to another	75%	6.3%	**91.7%**	8.3%
Specified time periods^1^	Periods of the day (e.g., hourly periods); days of the week (e.g., Monday-Friday); seasons (e.g., Winter, Summer)	68.8%	0%	**91.7%**	0%
Frequency^2^	Number of times an activity is performed within a specified time period (e.g., bouts/day)	**87.5%**	0%	**-**	**-**
Type^2^	The type of physical activity and/or sedentary behaviour being undertaken	75%	6.3%	**83.8%**	8.3%
***Activity pattern examples***					
Reporting the frequency, intensity and duration of activity bouts that occur throughout the day (e.g., daily number of minutes spent in ≥ 20-min moderate-intensity bouts; daily number of minutes spent in ≥ 30-min sedentary bouts)^1^		**81.3%**	12.5%	**-**	**-** **-**
Examining time spent in at least one intensity accumulated in a discrete time period during the day (e.g., lunchtime, recess)^1^		37.5%	50%	8.3%	66.7%
Reporting the frequency of postural transitions in specified time period(s) during the day (e.g., at work, during class time)^1^		75%	6.3%	**83.3%**	16.7%
Examining the time spent in least one intensity across different seasons (e.g., winter, summer)^1^		56.3%	25%	75%	8.3%
Examining the frequency, intensity and duration of activity bouts accumulated on different days of the week (e.g., weekday vs. weekend day, Monday vs. Tuesday)		75%	12.5%	**100%**	0%
Reporting the frequency, intensity and duration of activity bouts accumulated in discrete time period(s) during the day (e.g., during school time, during work time)		**81.3%**	0%	**-**	**-** -

*Percentage of participants reporting ‘Strongly agree’ or ‘Agree’

** Percentage of participants reporting ‘Strongly disagree’ or ‘Disagree’

^1^ Modified statement in Round 2 based on participant feedback

^2^ New statements in Round 2

Note: Bold % = 80% consensus achieved; - = Not assessed in Round 3 as consensus achieved in Round 2.

### Round 3

The Round 3 consisted of 10 statements (activity patterns and components = 5 statements; activity pattern examples = 4 statements; activity patterns reporting framework = 1 statement). Consensus (≥ 80%) was obtained for the activity pattern definition (83.3%), as well as the definitions of the activity components posture (92%), transition (92%), specified time periods (92%), and type (83%; Table 4). Two examples of activity patterns reached consensus (Table 4), and the one activity patterns reporting framework statement also obtained consensus (Table 3).

## Discussion

This modified Delphi study achieved consensus for defining activity patterns and activity pattern components. The agreed definition for activity patterns was: “The temporal structure of physical activity and sedentary behaviour [movement behaviours] accumulated over a specified time period during the waking hours” (consensus: 83.3%). Consensus was also achieved for the components of activity patterns, namely activity intensity, posture, activity bouts, transition, specified time periods, frequency, and type (Table 4). There was also consensus for four examples of activity patterns, which reflected the accumulation of activity bouts or postural transitions in specified time periods (e.g., during work time), as well as 10 components of a framework that can be used to guide the reporting of activity patterns within the literature (Supplementary files 2–4).

There has been considerable research that has examined the accumulation of activity in different age groups and populations [17, 28]. As the sophistication of methods for assessing activity of different intensities has developed, particularly the capabilities of device-based assessment to record data in real-time, the focus of activity patterns research has shifted from examining differences between males and females over time [29] or overall activity on weekdays and weekend days [30] to, for example, hourly patterns [31, 32] and the timing and duration of activity bouts [14, 37]. Such changes in approaches for assessing activity patterns over time may explain, to some extent, the lack of agreement that was demonstrated in a previous review as to how to measure and analyse activity patterns [15].

Previous reviews have highlighted that in the absence of a consistent definition of activity patterns, as well as inconsistency in the operationalisation and assessment of activity patterns, it has been difficult to draw conclusions about how activity is accumulated by different populations and how such patterns are associated with health and well-being [15, 17]. Examples of activity pattern components examined within the literature include sporadic and prolonged bouts of different movement intensities [13, 33], breaks in sitting time [34], postural transitions [35], tempo of activities [36], and time accumulated in different time periods of the day (e.g., hourly; [31]). In this Delphi study, such examples were considered in the operationalisation of activity pattern components rather than the definition of activity patterns. Such an approach therefore provides researchers with the discretion to use different measures, including self-report and device-based measures, that collect information on different components (e.g., activity bouts) under the broader activity pattern definition that reached consensus.

It is interesting to note that when examples of activity patterns that have been used in the literature were presented to participants, two examples did not achieve consensus (Table 4). These were “Examining time spent in at least one intensity accumulated in a discrete time period during the day (e.g., lunchtime, recess)” and “Examining the time spent in at least one intensity across different seasons (e.g., winter, summer)”. These specific proposed examples did not reflect the temporal nature of activity accumulation in the consensus definition of activity patterns in this study, despite such examples being historically described as patterns in previous literature [15]. This finding suggests that such historical research may not necessarily be classed as activity patterns research under this new agreed definition, particularly where comparisons between the volume of physical activity and sedentary behaviour accumulated at different time points (e.g., summer versus winter) or between different population groups has been examined without the inclusion of additional temporal patterns. This may have implications for future reviews synthesising evidence regarding associations between activity patterns and health and underpinning public health guidance.

It should be noted that this study did not aim to achieve consensus on how to define, for example, short or long activity bouts [13], or what time intervals would define a transition from one posture or intensity to another [35]. It is acknowledged that components such as bout lengths that have been examined vary within and between different age groups [17, 28, 37], and therefore general definitions for durations (e.g., short versus long) may not be realistic. This in turn may impact on understanding concerning how such activity pattern components are associated with health outcomes [17, 28]. As a result, the activity patterns reporting framework included statements that focused on providing detail about which activity pattern components had been investigated, how data had been processed, and a rationale for examining different bout lengths and time periods, where applicable. All the included statements in the reporting framework achieved consensus for inclusion, highlighting the importance of clearly describing the activity patterns that have been examined. It is therefore recommended that future studies use this framework to appropriately present their assessed activity patterns, as this will help standardise the consistency of reporting and improve comparability of study outcomes and facilitate the synthesis of evidence in the future.

In recent years, the term movement behaviours, which incorporates all behaviours that occur on a continuum from sleep to vigorous-intensity physical activity within a 24-hour period [38], has been increasingly used within the literature. Comments provided in the responses to this Delphi study highlighted the need to include movement behaviours within the definition of activity patterns, and to distinguish waking patterns from sleep patterns; the latter also having short- and long-term consequences for health and well-being across the lifespan [39, 40]. The definition of activity patterns includes physical activity and sedentary behaviour, identified as movement behaviours, to reflect advances in the literature. Moreover, the focus on the temporal structure during the waking day is consistent with previous definitions of sitting patterns [19, 41] and the assessment of physical activity patterns, though noting the considerable variability in how patterns were operationalised [15].

This study had several strengths. There was representation from experts located in different regions globally, though it is acknowledged that despite the authors’ efforts to recruit from varied areas, there was no representation from Asia and Africa. The participants consisted of early-, mid- and senior career researchers with expertise in activity patterns research. However, there are some limitations that should be noted. There was participant drop out across the study timepoints, which was greater than the anticipated 20% that has been documented by previous studies [18]. In addition, the response rate for Round 3 was low though the sample size (n = 12) is considered to be sufficient for obtaining consensus within Delphi studies [21, 24]. Due to a technical error in the survey, one item in the activity patterns reporting framework was not presented in the Round 3 survey, though consensus for inclusion was already achieved in the Round 1 survey. Snowball sampling was utilised to identify potential participants, which has been used in previous Delphi studies (e.g., [41]). However, it is possible that this may have introduced sampling bias into the study, particularly if participants held similar views on the definition of an activity pattern or pattern component. Lastly, the framework for reporting activity patterns is intended to be applicable for research that has used both subjective and device-based measures to assess activity patterns, though some items are more focused on device-based measures. Whilst the framework may improve the consistency of reporting within the literature, it is not intended to standardise and harmonise data reduction and analytical methods.

## Conclusion

This Delphi study resulted in achieving a consensus on the definition of activity patterns and activity patterns components for the first time. Additionally, this study also developed a framework for reporting activity patterns via a consensus approach. Given the significant interest in quantifying how individuals accumulate their physical activity and sedentary behaviour across the lifespan to inform the development of public health guidelines and interventions efforts, it is hoped that this consensus definition and reporting activity patterns framework will guide future research and facilitate the consistent reporting of activity patterns.

## Electronic supplementary material

Below is the link to the electronic supplementary material.

Supplementary Material 1: Table S1: Summary of adaptations to items between Round 1 and Round 2.

Supplementary Material 2: Table S2: Final definition of activity patterns and activity pattern components (≥ 80% consensus achieved).

Supplementary Material 3: Table S3: Final examples of activity patterns (≥ 80% consensus achieved).

Supplementary Material 4: Table S4: Activity Patterns Reporting Framework (≥ 80% consensus achieved).

Supplementary Material 5: STROBE Checklist for observational studies

## Data Availability

The datasets generated and analysed during the current study are not publicly available due to ethics board requirements but are available from the corresponding author on reasonable request and pending approval from the relevant ethics committees.

## List of abbreviations

Min: Minute/s
