# Supplementary material for: Defining and reporting activity patterns: a modified Delphi study
Source: Int J Behav Nutr Phys Act. 2023 Jul 25;20:89. doi: 10.1186/s12966-023-01482-6 (PMC10367379; doi:10.1186/s12966-023-01482-6)
Supplement: Supplementary file 1 — Supplementary Material 1: Table S1: Summary of adaptations to items between Round 1 and Round 2. [file 12966_2023_1482_MOESM1_ESM.docx]

**Table S1: Summary of adaptations to items between Round 1 and Round 2**

| **Component or example** | **Item included in Round 1** | **Change made** | **Adapted item in Round 2** |
| --- | --- | --- | --- |
| ***Activity patterns and components definitions*** | |  |  |
| Activity pattern definition | How and/or when an individual accumulates waking activity of at least one intensity and/or posture. | Participant/s recommended focusing on temporal nature of activity accumulation. | The temporal structure of physical activity and sedentary behaviour [movement behaviours] accumulated over a specified time period during the waking hours. |
| Activity intensity definition | The energy expended while engaging in different activities or behaviours. | Participant/s recommended revising to focus on rate of energy expenditure for waking behaviours. | Rate of energy expenditure required to perform waking activities. |
| Posture definition | The position of the body while lying, sitting, or upright. | Participant/s recommended replacing position with posture. | The posture of the body (e.g., lying, reclining, sitting, or upright). |
| How activity is accumulated definition | The frequency and duration of activity bouts and/or the transition between bouts of different intensities or postures. | No change was made as participants recommended the item should be dropped. | Exclusion of item reached consensus. |
| Activity bout definition | Unbroken period of time engaged in physical activity and/or sedentary behaviour. | No changes made. | No change. |
| Transition definition | Change from one activity intensity or posture to another. | No changes made. | No change. |
| When activity is accumulated | Periods of the day in which activity of at least one intensity and/or posture occurs (e.g., hourly periods, class time, recess, before work, during work) and/or days of the week (e.g., Monday-Sunday, workday vs non-workday). | Participant/s recommended focusing on specified time periods. | Periods of the day (e.g., hourly periods); days of the week (e.g., Monday-Friday); seasons (e.g., Winter, Summer) |
| Frequency definition | Not included. | Recommended for inclusion by participant/s. | Number of times an activity is performed within a specified time period (e.g., bouts/day) |
| Type | Not included. | Recommended for inclusion by participant/s. | The type of physical activity and/or sedentary behaviour being undertaken |
| ***Activity pattern examples*** | |  |  |
| Frequency, intensity and duration of activity bouts that occur throughout the day (e.g., minutes spent in 20-min sedentary bouts). | | Participant/s recommended thew inclusion of physical activity and sedentary behaviour examples. | Reporting the frequency, intensity and duration of activity bouts that occur throughout the day (e.g., daily number of minutes spent in ≥20-min moderate-intensity bouts; daily number of minutes spent in ≥30-min sedentary bouts). |
| Total volume of at least one intensity accumulated in discrete time period(s) during the day (e.g., recess, lunchtime, after school). | | Participant/s recommended using the phrase “time spent in…”. | Examining time spent in at least one intensity accumulated in a discrete time period during the day (e.g., lunchtime, recess). |
| Frequency of postural transitions in discrete time period(s) during the day (e.g., at work, during class time). | | “Reporting the…” included for consistency with other items. No changes to the item itself. | Reporting the frequency of postural transitions in specified time period(s) during the day (e.g., at work, during class time). |
| Total volume of at least one intensity accumulated across different seasons (e.g., winter, summer). | | Participant/s recommended using the phrase “time spent in…”. | Examining the time spent in least one intensity across different seasons (e.g., winter, summer). |
| Frequency, intensity and duration of activity bouts accumulated on different days of the week (e.g., weekday vs weekend day). | | Participant/s recommended including additional examples for days of the week. | Examining the frequency, intensity and duration of activity bouts accumulated on different days of the week (e.g., weekday vs weekend day, Monday vs Tuesday). |
| Frequency, intensity and duration of activity *bouts* accumulated in discrete time period(s) during the day (e.g., during school time, during work time). | | “Reporting the…” included for consistency with other items. No changes to the item itself. | Reporting the frequency, intensity and duration of activity bouts accumulated in discrete time period(s) during the day (e.g., during school time, during work time). |
| ***Activity Patterns Reporting Framework*** | |  |  |
| The activity intensity (or intensities) and/or posture(s) being investigated should be clearly defined and reported. | | No changes made. | The activity intensity (or intensities) and/or posture(s) being investigated should be clearly defined and reported. |
| An explanation of how the activity pattern is defined/derived should be clearly reported (e.g., minimum bout length, interruptions to sedentary time, %tolerance in other  intensity/posture permitted, break rate). | | No changes made. | An explanation of how the activity pattern is defined/derived should be clearly reported (e.g., minimum bout length, interruptions to sedentary time, %tolerance in other  intensity/posture permitted, break rate). |
| A rationale for the choice of activity bout(s) length(s) and/or  transitions should be reported, where applicable (e.g., bouts based on previous research, bouts are data driven). | | No changes made. | A rationale for the choice of activity bout(s) length(s) and/or  transitions should be reported, where applicable (e.g., bouts based on previous research, bouts are data driven). |
| The way in which activity bouts and/or transition data are defined and analysed should be clearly reported (e.g., median number of breaks, minutes spent in [specified intensity] bout, frequency of bouts, usual bout duration, alpha, Gini, fragmentation index, contribution of bouts to daily volume, etc). | | No changes made. | The way in which activity bouts and/or transition data are defined and analysed should be clearly reported (e.g., median number of breaks, minutes spent in [specified intensity] bout, frequency of bouts, usual bout duration, alpha, Gini, fragmentation index, contribution of bouts to daily volume, etc). |
| The time period(s) and/or days of interest should be clearly defined (e.g., schooltime, at work, hourly periods, average day etc). | | No changes made. | The time period(s) and/or days of interest should be clearly defined (e.g., schooltime, at work, hourly periods, average day etc). |
| A rationale for the choice of any specific time period(s) and/or days of interest should be clearly provided. | | No changes made. | A rationale for the choice of any specific time period(s) and/or days of interest should be clearly provided. |
| The outcome variables for the time period(s) and/or days should be clearly reported (e.g., volume of activity accumulated in hourly periods on weekdays and weekend days, frequency of  transitions during work, etc) | | No changes made. | The outcome variables for the time period(s) and/or days should be clearly reported (e.g., volume of activity accumulated in hourly periods on weekdays and weekend days, frequency of  transitions during work, etc) |
| The outcome variables for the time period(s) and/or days should be clearly reported (e.g., volume of activity accumulated in hourly periods on weekdays and weekend days, frequency of  transitions during work, etc) | | No changes made. | The outcome variables for the time period(s) and/or days should be clearly reported (e.g., volume of activity accumulated in hourly periods on weekdays and weekend days, frequency of  transitions during work, etc) |
| Not included. | | Recommended for inclusion by participant/s. | The processing of activity patterns data should be clearly reported |
| Not included. | | Recommended for inclusion by participant/s. | A rationale for choosing and defining specific activity pattern components should be reported, where applicable |
