# Supplementary material for: Defining and reporting activity patterns: a modified Delphi study
Source: Int J Behav Nutr Phys Act. 2023 Jul 25;20:89. doi: 10.1186/s12966-023-01482-6 (PMC10367379; doi:10.1186/s12966-023-01482-6)
Supplement: Supplementary file 2 — Supplementary Material 2: Table S2: Final definition of activity patterns and activity pattern components (≥ 80% consensus achieved). [file 12966_2023_1482_MOESM2_ESM.docx]

**Table S2: Final definition of activity patterns and activity pattern components (≥80% consensus achieved)**

| **Term** | **Definition** |
| --- | --- |
| Activity patterns | The temporal structure of physical activity and sedentary behaviour [movement behaviours] accumulated over a specified time period during the waking hours |
| Activity intensity | Rate of energy expenditure required to perform waking activities |
| Posture | The posture of the body (e.g., lying, reclining, sitting, or upright) |
| Activity bout | Unbroken period of time engaged in physical activity and/or sedentary behaviour [33,37] |
| Transition | Change from one activity intensity or posture to another |
| Specified time periods | Periods of the day (e.g., hourly periods); days of the week (e.g., Monday-Friday); seasons (e.g., Winter, Summer) |
| Frequency | Number of times an activity is performed within a specified time period (e.g., bouts/day) |
| Type | The type of physical activity and/or sedentary behaviour being undertaken |
