# Supplementary material for: Defining and reporting activity patterns: a modified Delphi study
Source: Int J Behav Nutr Phys Act. 2023 Jul 25;20:89. doi: 10.1186/s12966-023-01482-6 (PMC10367379; doi:10.1186/s12966-023-01482-6)
Supplement: Supplementary file 3 — Supplementary Material 3: Table S3: Final examples of activity patterns (≥ 80% consensus achieved). [file 12966_2023_1482_MOESM3_ESM.docx]

**Table S3: Final examples of activity patterns (≥80% consensus achieved)**

| **Activity pattern example** |
| --- |
| Reporting the frequency, intensity and duration of activity bouts that occur throughout the day (e.g., daily number of minutes spent in ≥20-min moderate-intensity bouts; daily number of minutes spent in ≥30-min sedentary bouts) |
| Reporting the frequency of postural transitions in specified time period(s) during the day (e.g., at work, during class time) |
| Examining the frequency, intensity and duration of activity bouts accumulated on different days of the week (e.g., weekday vs weekend day, Monday vs Tuesday) |
| Reporting the frequency, intensity and duration of activity bouts accumulated in discrete time period(s) during the day (e.g., during school time, during work time) |
