# Supplementary material for: Defining and reporting activity patterns: a modified Delphi study
Source: Int J Behav Nutr Phys Act. 2023 Jul 25;20:89. doi: 10.1186/s12966-023-01482-6 (PMC10367379; doi:10.1186/s12966-023-01482-6)
Supplement: Supplementary file 4 — Supplementary Material 4: Table S4: Activity Patterns Reporting Framework (≥ 80% consensus achieved). [file 12966_2023_1482_MOESM4_ESM.docx]

**Table S4: Activity Patterns Reporting Framework (≥80% consensus achieved)**

| **Item #** | **Statement** |
| --- | --- |
| 1 | The activity intensity (or intensities) and/or posture(s) being investigated should be clearly defined and reported |
| 2 | An explanation of how specific activity pattern components are defined/derived should be clearly reported |
| 3 | A rationale for examining activity bout(s) and/or transitions should be reported, where applicable |
| 4 | The way in which activity bouts and/or transition data are defined and analysed should be clearly reported, where applicable |
| 5 | The time period(s) and/or days of interest should be clearly defined, where applicable. |
| 6 | A rationale for the choice of any specific time period(s) and/or days of interest should be clearly provided. |
| 7 | The outcome variables for the time period(s) and/or days should be clearly reported |
| 8 | The method used to assess activity patterns should be clearly reported |
| 9 | The processing of activity patterns data should be clearly reported |
| 10 | A rationale for choosing and defining specific activity pattern components should be reported, where applicable |
